# Urine-derived induced pluripotent/neural stem cells for modeling neurological diseases

**DOI:** 10.1186/s13578-021-00594-5

**Published:** 2021-05-13

**Authors:** Tianyuan Shi, Martin Cheung

**Affiliations:** School of Biomedical Sciences, Li Ka Shing Faculty of Medicine, The University of Hong Kong, Hong Kong, China

**Keywords:** Urine-derived stem cells, Neurological diseases, Induced pluripotent stem cells, Induced neural stem cells

## Abstract

Neurological diseases are mainly modeled using rodents through gene editing, surgery or injury approaches. However, differences between humans and rodents in terms of genetics, neural development, and physiology pose limitations on studying disease pathogenesis in rodent models for neuroscience research. In the past decade, the generation of induced pluripotent stem cells (iPSCs) and induced neural stem cells (iNSCs) by reprogramming somatic cells offers a powerful alternative for modeling neurological diseases and for testing regenerative medicines. Among the different somatic cell types, urine-derived stem cells (USCs) are an ideal cell source for iPSC and iNSC reprogramming, as USCs are highly proliferative, multipotent, epithelial in nature, and easier to reprogram than skin fibroblasts. In addition, the use of USCs represents a simple, low-cost and non-invasive procedure for generating iPSCs/iNSCs. This review describes the cellular and molecular properties of USCs, their differentiation potency, different reprogramming methods for the generation of iPSCs/iNSCs, and their potential applications in modeling neurological diseases.

## Introduction

Neurological diseases are caused by a malfunction of the central and peripheral nervous systems. They can be caused by a multitude of factors including but not limited to genetic mutations, trauma, malnutrition, bacterial infection, and/or environmental factors. The exploration of the pathogenesis of complex neurological diseases can be hampered by the limited accessibility to nervous tissues from patients. To overcome these limitations, rodents have been commonly used as surrogate models of human neurological diseases to study disease pathogenesis and to evaluate the efficacy and safety of pre-clinical drugs. However, rodents and humans differ greatly in terms of genetics, metabolism, and physiology, making animal models less than perfect for modeling human diseases.

Human embryonic stem cells (hESCs) possess inherent self-renewal capacity and pluripotent potential to differentiate into any cell type of the three germ layers (ectoderm, mesoderm, and endoderm), which offers an alternative to animal models in neurological research. Several studies have successfully demonstrated the differentiation of hESCs into neural stem cells/progenitors, neuronal subtypes [1, 2], oligodendrocytes [3], and astrocytes [4], which can be potential cell sources for modeling and developing therapeutics for several neurological diseases such as amyotrophic lateral sclerosis (ALS) [5–7] and spinal muscular atrophy (SMA) [8, 9]. Although there are several established methods for deriving hESCs from the inner cell mass of blastocyst, there are several caveats to using hESCs for diseases modeling: 1) Isolation of hESCs involves destroying human embryos (or blastocyst) that raises ethical concerns, 2) hESC-derived cells are allogeneic and cause immune rejection in recipient transplantation therapies, and 3) hESCs cannot fully recapitulate the genetic background and phenotypic readouts of complex or sporadic diseases with no well-defined genetic etiology.

The breakthrough discovery of induced pluripotent stem cells (iPSCs) by Shinya Yamanaka provides a better source of pluripotent stem cells for modeling and treating neurological diseases. Somatic cells can be reprogrammed into iPSCs by the induction of *Oct3/4*, *Sox2*, *Klf4*, and *c-Myc* (OSKM) genes via virus-mediated delivery [10]. Patient-derived iPSCs not only provide diseases-specific genetic information, but can also avoid immune rejection in transplantation therapies. There are also no ethical issues, as autologous cells are used to generate iPSCs. Patient-specific iPSCs exhibit similar molecular profiling and pluripotent potential to that of hESCs, thus iPSCs can serve as an excellent disease modeling platform to study the molecular mechanisms underlying various neurological diseases such as Huntington’s disease [11], Alzheimer's disease (AD) [12, 13], ALS [14] [15] and SMA [16–18].

In addition, direct lineage reprogramming can be used to generate induced neural stem cells (iNSCs) from somatic cells providing a rapid, efficient, and safer approach without needing to go through the pluripotent state, which risks tumor formation in transplantation therapy [19–21]. Although skin fibroblasts [22] and blood cells [23] have been commonly employed for reprogramming into iPSCs and iNSCs, the procedures to obtain them are invasive and sometimes painful, in particular skin biopsies can cause potential complications in patients [24] and so obtaining cell samples from patients especially young children with rare diseases is difficult. This can be avoided by repeated urine collections without medical assistance. In recent decades, urine-derived stem cells (USCs) have garnered much interest for the generation of iPSCs and iNSCs, as they have several advantages: (1) non-invasive, ease of collection and isolation to establish low cost culture system without special substrates, (2) high expandability (doubling time (DT): 20-28 hours) compared with other widely used adult stem cells or progenitors such as bone marrow-derived mesenchymal stem cells (BMSCs) (DT: 3.5 - 6.73 days) and peripheral blood mononuclear cells (PBMCs) (DT: 55–62 hours) [25–27], (3) better adipogenic and endothelial abilities as well as vascularization potential compared to BMSCs and placenta decidua basalis-derived mesenchymal stem cells (PDB-MSCs) [28], (4) once isolated, USCs do not involve complicated methods of sample processing compared to PBMC which involves the difficult and tedious isolation process of CD34+ cells from peripheral blood [29], (5) higher reprogramming efficiency due to an epithelial origin of USCs which do not require mesenchymal-to-epithelial transition (MET) during reprogramming, unlike skin fibroblasts, (6) no ethical issues, (7) low immunogenicity, and (8) absence of tumorigenicity with normal karyotype [30–33]. Therefore, USCs can serve as an ideal cell source for disease modeling and for developing treatment options.

In this review, we discuss the main characteristics of USCs, the different approaches for reprogramming them into iPSCs and iNSCs, and their applications in modeling neurological diseases.

## Characteristics of USCs

Each human kidney is composed of more than 1 million nephrons that filter about 113–144 L of blood and generate 0.94 to 1.8 L of urine every day. Approximately 2000–7000 cells detach from the urinary system daily and are excreted in the urine [32]. A subpopulation of these cells (USCs) has stem cell properties including high proliferative capacity, molecular expression profile, multipotency, and immunomodulatory properties. Together with their low cost and non-invasive sampling, these USCs offer significant advantages over other somatic cells, such as skin fibroblasts, blood, and bone marrow-derived mesenchymal stem cells, for iPSCs and lineage reprogramming. Here, we discuss the isolation, proliferation, cell surface markers, and differentiation potency of USCs, as well as their therapeutic potential for various applications.

### Isolation and proliferation of USCs

The first study describing the collection of exfoliated urinary cells was reported by Sutherland and Bain in 1972 [34]. They successfully obtained proliferative cell populations from the urine of four infants less than 2 days old. Zhang et al*.* [35] was first to report the presence of renal progenitors in cultured urine-derived cells from 15 healthy people and 8 patients with vesicoureteral reflux. They identified fully differentiated cells, differentiating cells, and progenitor-like cells based on their proliferative capacity and differentiation potency. There were about 5.6 × 10^3^ living cells per 100 mL of urine, but the majority of them (99%) were fully differentiated cells with large and flat appearance that could not attach to the culture plates. There were only 1–2 differentiating cells per 100 mL of urine, which attached to culture dish and expand to 10^3^ within 2 ~ 3 weeks, but did not grow after passage. There were about 2–7 progenitor-like cells per 100 mL of urine with ability to form a uniform, condensed colony from a single cell in 2 weeks. These cells could grow for 8 passages in vitro and differentiate into urothelial, smooth muscle, endothelial, and interstitial cells. However, the proportion of cells expressing stem/progenitor markers decreased after each passage. The success rate of isolating progenitor-like cells from USCs was higher in males than females (70% vs. 42%) [36], probably due to the urinary tract differences between male and female, resulting in more frequent microorganism contamination during urine sample collection from female.

The collection procedure of USCs is simple, quick, and reproducible. Briefly, 100–300 mL of voided midstream urine was collected from donors in sterilized containers. After centrifugation, the sediments were washed with phosphate buffered saline (PBS) containing antibiotics, and cell pellets were then resuspended in medium and cultured in gelatin-coated 24-well plates [37, 38]. Despite slight variations in the composition of the culture media between different studies, the core media components included fetal bovine serum, human epidermal growth factor, insulin, transferrin, adrenaline, triiodothyronine, and L-glutamine.

**Fig. 1:**
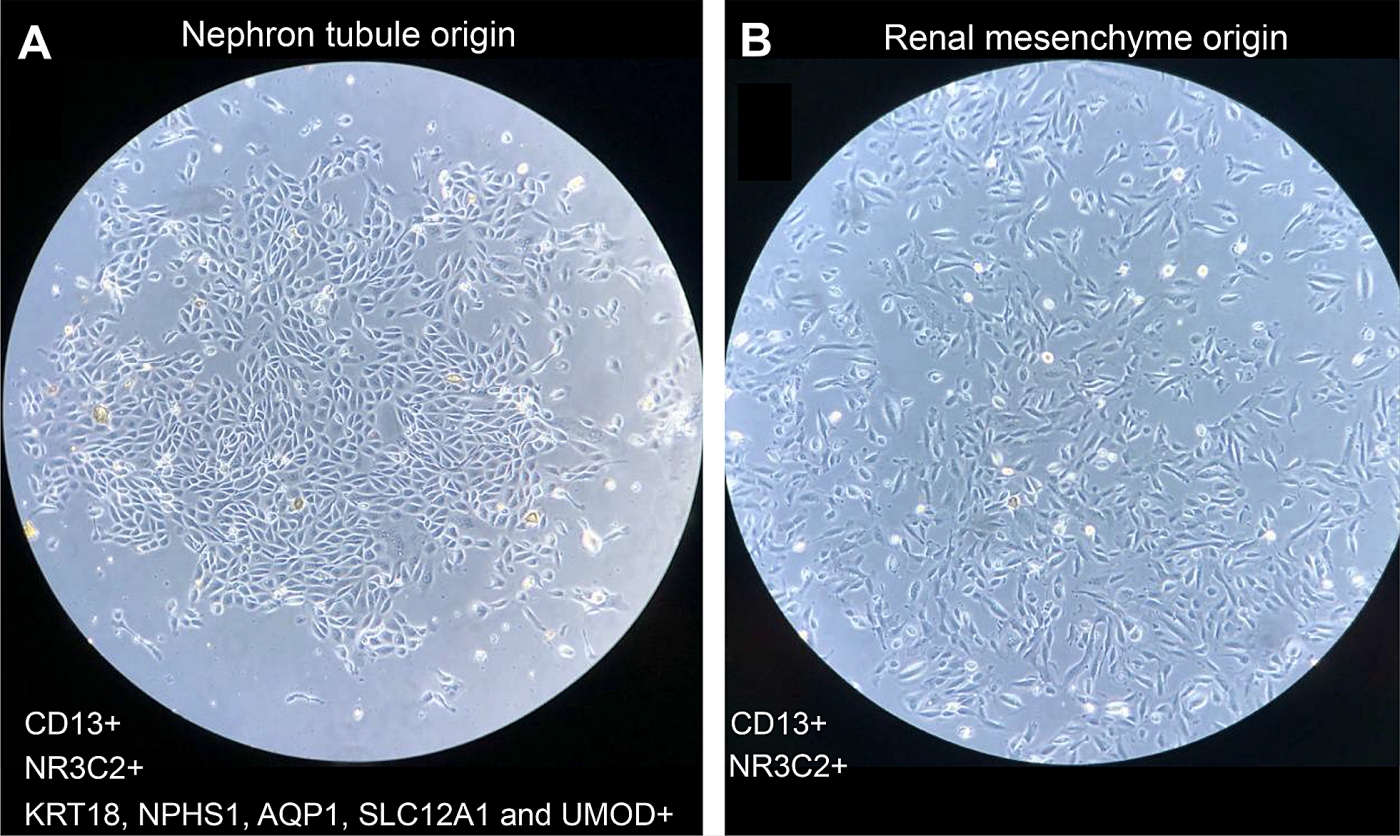
Different morphologies of type I **a** and type II **b** USCs cultured on gelatin on day 15 after seeding

Several types of cells were found in the voided urine. Differentiated squamous cells and blood cells that did not attach to the culture plate were removed after the first medium refresh. A study by Doerrenhaus [39] compared the cells in urine sediments from healthy people and in urine directly collected from the renal pelvis of urological patients, which found no difference in the cell morphologies between the two collection approaches. Besides, urine cells from urinary tract express urothelial cells marker cytokeratin-7 (CK7) [40], whereas cells from renal system express renal epithelial marker carbonic anhydrase [39]. Furthermore, urine cells from the renal system showed two different morphologies consisting of cobblestone-like type I and spindle-like type II cells. Both cell types could be isolated from the same individual, with type I cells more frequently obtained compared to type II cells. Type I cells were more regular in shape with smooth-edged contours and can form domes (hemicysts), whereas type II cells were randomly arranged and did not form domes (Fig. 1). Type I cobblestone-like cells were thought to be originated from nephron tubule, while type II spindle-like cells originated from renal mesenchyme based on their differential expression of markers for various parts of nephrons (see below) [39, 41]. Type II cells were also found to have higher proliferative capacity and could be cultured up to passage 10, whereas type I cells had less proliferative capacity and entered senescence around passage 5 [32].

Urine colonies were generally visible between 3 and 5 days after the initial seeding, reaching 80%-90% confluence in 15–20 days. After the first subculture, cell counting kit-8 assay revealed the USCs typically displayed an S-shape growth curve, which began in a stationary phase during the first 1–2 days and reached a rapid growth rate from 3–4 days, followed by a slow growth rate at 5–6 days [38]. Moreover, karyotyping analysis indicated normal number, size, and shape of chromosomes without tumorigenic phenotypes after repeated passages of USCs [38]. It was reported that USCs from children showed a lower tendency to undergo senescence than samples from middle-aged or older groups, as determined by senescence-associated β galactosidase staining, although USCs from all age groups showed high proliferative ability, with potential for use in tissue engineering applications [31, 38].

### Cell surface properties and differentiation potency of USCs

Cell surface markers such as clusters of differentiation (CD) have been widely used to identify and characterize different types of stem cells. The USCs expressed pluripotent embryonic stem cell (ESC) markers, TRA-1-60, TRA-1-81, SSEA4, SOX2, OCT3/4, c-MYC, and KLF4 [42]. Renal progenitor surface markers such as CD13, CD24, CD90, and CD133 were also present in the USCs [41], indicating they originated in the kidney. The expression of mesenchymal stem cells (MSCs) surface markers, including CD29, CD73, CD90, CD105, CD166, and STRO-1, also suggests USCs could be considered as a source of MSCs. However, they did not express hematopoietic stem cell markers such as CD11b, CD14, CD31, CD34, CD45, and CD309, which preclude a hematopoietic origin [25, 35, 38, 43, 44]. In addition, a subpopulation of USCs were found to be positive for markers characteristic of pericytes (CD146, NG2, and PDGF-rβ), endothelial cells (vWF, CD31 and CD146), epithelial and smooth muscle cells (α-SMA, Desmin), and elongated interstitial cells (c-Kit) [25, 42, 43]. Both types I and II USCs showed positive for mesenchyme marker vimentin [41] and renal epithelial markers like CD13 and NR3C2 [45, 46]. Type 1 cells were positive for markers characteristic of urogenital epithelium (KRT18), podocyte (NPHS1), proximal convoluted tubule cell (AQP1), the loop of Henle cell (SLC12A1), and distal convoluted tubule cell (UMOD), but few were positive for AQP2 (marker for collecting duct cells), indicating their cellular origin from the nephron tubules which include the Bowman’s capsule to the distal convoluted tubule, but not from the collecting duct. In contrast, type II cells were weakly positive for SLC12A1 and UMOD, and negative for the rest of the markers, suggesting their renal mesenchymal origin near the loop of Henle and the distal convoluted tubule [41].

A single clone of USCs could give rise to a large population with multipotent potential with 60–70 population doublings. Upon induction with appropriate media in vitro, one single USC could potentially differentiate into endothelial, osteogenic, chondrogenic, adipogenic, skeletal, myogenic, and neurogenic lineages [42, 47, 48]. As USCs originate from the upper urinary tract that has ectodermal epithelial stem cell potency and MSC surface properties, USCs exhibit higher differentiation potential for myogenic, neurogenic, and endothelial lineages compared to adipose-derived stem cells (ADSCs). Indeed, 3 weeks after transplantation of GFP-labeled USCs into rat motor cortex, they expressed neuronal markers, β-III-tubulin and Nestin, and astrocyte marker GFAP, indicating they had committed to neural lineages [38]. The neurogenic potency of USCs was further supported by using a cocktail of small molecules to drive the differentiation into GABAergic [49] and glutamatergic neurons [50]. In contrast, USCs were less effective at undergoing adipogenic, osteogenic, and chondrogenic differentiation than ADSCs, which originate from the mesoderm and have a stronger potency to give rise to mesenchymal cell types including adipocytes, osteoblasts, and chondrocytes [51]. Furthermore, USCs possessed telomerase activity, which maintained telomere length over several passages with normal karyotype. The USCs did not form teratomas, even 3 months after renal subcapsular cell implantation, despite harboring ES-like properties [35]. Importantly, USCs do not express human leucocytes antigen (HLA)-DR glycoproteins, which are commonly found in antigen-presenting cells and are responsible for triggering immune responses [38, 42, 44, 51]. Distinguishable from ESCs and other multipotent cell types, USCs exhibited various cell surface markers that conferred them with high proliferative capacity, broader differentiation potential, and immunosuppressive properties, which are favorable for transplantation applications. The expressions of different cell surface markers in USCs are listed in Table 1.

**Table 1:** The expression of cell surface markers in USCs

Surface markers in USCs		% of expression	References
Embryonic stem cell markers	TRA-1–81	±	[42]
	TRA-1–60	±	[42]
	SSEA4	+	[42]
Epithelial markers	E-cadherin	+	[32, 52]
	β-catenin	+	[32, 52]
	Occludin	+	[32]
	Claudin 1	+	[32]
	ZO-1	+	[32, 52]
	KRT7	+	[8, 32, 52]
	KRT14	+	[53]
	KRT15	+	[53]
	KRT16	+	[53]
	KRT18	+ (type I),—(type II)	[41]
	KRT19	+	[53]
	CD326	+	[53]
Epithelial basal markers	CD44	+	[25, 38, 54]
Renal epithelial markers	CD13	+	[23, 32, 44, 52]
	L1CAM	+	[52]
	NR3C2	+	
	SLC2A1	+	
	CD24	+	[41]
	CD29	+	
	CD34	−	
	CD73	+	
	CD90	+	
	CD105	+	
	CD133	±	
	UMOD	+ (type I),—(type II)	
	NPHS1	+ (type I),—(type II)	
	AQP1	+ (type I),—(type II)	
	AQP2	−	
	SLC12A1	+ (type I),—(type II)	
Mesenchymal stem cells markers	SSEA-4	+	[25, 55]
	CD29	+	[38, 44]
	CD73	+	[25, 31, 38, 55]
	CD90	+	[31, 38, 44]
	CD105	±	[25, 31, 38, 44]
	CD166	+	[44]
	STRO-1	+	[25]
Fibroblast markers	Actin	+	[32, 52]
	Vimentin	±	[32, 52]
	Fibronectin	±	[32, 52]
	Twist 1	−	[32]
	Slug	−	[32]
Pericyte markers	CD146	± , +	[25, 31, 42]
	NG2	±	[25, 42]
	PDGF-rβ (CD 140b)	±	[25, 42]
Hematopoietic stem cells markers	CD11b	−	[42, 56]
	CD14	−	[56]
	CD31	−	[25]
	CD34	−	[25, 31, 38, 44]
	CD45	-, ±	[25, 31, 44, 54]
	CD133	±	[25, 38]
	CD309	−	[38]
	HLA-ABC (MHC-1)	+	[42, 57]
	HLA-DR (MHC-II)	−	[38, 42, 44, 51]

**−**: percentage of expression < 1%, ± : 1% < percentage of expression < 50%, + : percentage of expression > 50%

## Generation of iPSCs and iNSCs from USCs

As USCs can be easily collected in voided urine and have high expandability for at least five passages with reprogramming capacity, they could serve as an ideal cell source for iPSCs and iNSCs generation. Different reprogramming strategies have been used for the generation of iPSCs and iNSCs from various somatic cell types, and different methods have different pros and cons [58]. Strategies include integrating retroviruses and lentiviruses, and non-integrating methods such as Sendai virus, episomal plasmids, and small molecules. Here, we discuss different reprogramming methods used to generate USC-derived iPSCs (UiPSCs) and iNSCs (UiNSCs).

### UiPSCs reprograming

#### Retroviruses

In 2011, Zhou et al*.*[32, 52] first used retroviruses expressing OSKM factors to infect USCs with iPSC reprogramming efficiency between 0.1% and 4% based on the number of alkaline phosphatase-positive clones with hESC morphology. The USCs transduced at later passages exhibited decreasing reprogramming efficiency from 0.3% to 3% at passage 2 to only 0.05% at passage 4, which indicates the USCs should be used for iPSC reprogramming before passage 4. These Urine iPSC (UiPSCs) colonies were positive for pluripotent markers such as Sox2, Oct4, NANOG, TRA-1-60, TRA-1-81, SSEA-3, and SSEA-4, and had silenced retroviral expression of Sox2, Oct4, Klf4 and c-Myc at the end of reprogramming. DNA microarrays showed hESCs (H9) and UiPSCs had similar global gene expression profiles. The UiPSCs also had normal karyotype and demethylation of the proximal Oct4 and Nanog promoters. The pluripotency of UiPSCs were demonstrated by teratoma and embryoid body (EB) assays, which revealed they could differentiate into all three germ layers. In addition, UiPSCs could be directed to differentiate into neural cells, hepatocytes, and cardiomyocytes. The retroviral-mediated OSKM reprogramming approach has been successfully employed to generate patient-specific UiPSCs for modeling of paroxysmal kinesigenic dyskinesia and X-linked Danon disease [59, 60].

#### Lentivirus

A recent study used lentivirus expressing OSKM to generate UiPSCs, which expressed typical hESC markers and demonstrated pluripotency in vitro and in vivo [61]. Several patient-specific UiPSCs have been generated by lentiviruses for modeling diseases such as systemic lupus erythematosus [62], cryptorchid [63], and spinal muscular atrophy [17]. Compared to retroviruses that infect dividing cells, lentiviruses can infect both dividing and non-dividing cells with larger cloning capacity. Although both viruses have similar reprogramming efficiency, lentiviral-mediated expressions of transcription factors were not silenced following reprogramming. This can be resolved by using a doxycycline-inducible lentivirus to control the expression of reprogramming factors in a time-dependent manner [64]. However, a drawback of using integrated viruses is their non-specific integration into different genomic sites in each iPSC clone, resulting in unavoidable heterogeneity between clones [65, 66]. The different viral integration sites could affect the efficiency and interfere with the expression levels of reprogramming factors or even other tumor suppressor genes and/or oncogenes, potentially leading to tumor formation upon transplantation of infected cells [65]. Therefore, using retroviruses or lentiviruses for the delivery of reprogramming factors is not considered safe for therapeutic applications.

#### Sendai virus and episomal vector

Another widely used method for iPSC reprogramming is Sendai virus, which is a single stranded negative sense RNA virus that does not integrate into the genome. Sendai reprogramming has been used to generate UiPSCs from the urine of patients with attention deficit hyperactivity disorder (ADHD) [67], obsessive–compulsive disorder [68], Duchenne muscular dystrophy [69–71], dilated cardiomyopathy [72], heterozygous PAI-I mutation [73], ventricular septal defect (VSD) [74], X-linked Alport syndrome (X-LAS) [75], spinal cord injury [76], type 2 diabetes mellitus [77], and healthy donors [78]. The majority of the iPSC colonies were undetectable for Sendai virus transgenes after 7 passages [67]. Although the Sendai virus is integration free, there is still a risk that Sendai RNA is retained in the first passage of iPSC lines [79].

Episomal induction is another frequently used integration-free iPSCs reprogramming method, which can circumvent these problems. In 2013, Xue et al*.* [80] generated stable iPSC colonies from USCs transfected with the Epstein-Barr virus-encoded nuclear antigen-1-oriP episomal vectors expressing OCT4, SOX2, SV40T, and KLF4 with the miR-302–367 cluster by electroporation. The miR-302 family, which is specifically expressed in ESCs, can enhance reprogramming efficiency [81], and activate Ink4a and Arf to inhibit tumorigenicity in hESCs by targeting the oncogene *Bmi1* [82]. The resulting UiPSCs did not exhibit exogenous reprogramming factors or episomal backbones, and no insertion mutations were found. Moreover, the lack of oncogene *c-MYC* together with feeder-free and serum-free medium further reduced the chance of tumorigenicity, which paves the way for future GMP generation of clinical grade non-viral human iPSCs. In addition, the epithelial nature of USCs makes it easier to reprogram them into iPSCs compared to mesenchymal skin fibroblasts, which need to undergo MET to obtain a pluripotent state [83]. The presence of high levels of epithelial markers or accelerators of MET, such as E-CADHERIN, CLAUDIN, OCCLUDIN, miR-200c, and miR-302b, but low levels of MET repressor Twist facilitated the reprogramming efficiency of USCs into iPSCs [80]. The episomal approach has been used to generate UC lines from 20 individuals with diverse genetic and disease backgrounds including ALS, Parkinson’s disease, β-thalassemia, and Hemophilia A [80]. Another study investigated PCSK9-mediated autosomal dominant hypercholesterolemia (ADH) using UiPSCs generated by episomal vectors coding for OCT4, SOX2, KLF4, MYC, LIN28, NANOG and SV40LT, and a non-episomal vector coding for miR302/367 [43]. In addition, integration-free UiPSC lines from individuals with Down syndrome (DS) [84], Phenylketonuria (PKU) [73] and Type 2 long QT syndrome [85] have been successfully established via episomal technology.

#### Small molecules

Most of the episomal induction approaches employ at least one tumorigenic factor, such as c-Myc, SV40-LT, or p53 inhibitors, and other factors to facilitate somatic reprogramming, but this could cause tumorigenesis in iPSCs. To circumvent this issue, a low-risk 6F/BM1-4C reprogramming system containing six factors (L-Myc, Sox2, Oct4, Glis1, Klf4, and miR-302 cluster) in the episomal vector and the four compounds (inhibitor of lysine-demethylase1, methyl ethyl ketone, glycogen synthase kinase 3β, and histone deacetylase) were able to generate UiPSCs efficiently within a short period of time [86]. These UiPSCs exhibited reduced chromosomal variation and higher genomic stability compared to iPSCs induced by conventional episomal vectors.

Due to the heterogeneity of USCs, a substantial portion show poor proliferative ability, making it difficult to use non-viral approaches for iPS reprogramming. A recent report identified a cocktail containing cyclic pifithrin-α (a P53 inhibitor), A-83–01, CHIR99021, thiazovivin, NaB, and PD0325901 that significantly enhanced the reprogramming efficiency in USCs with low proliferation. Instead of culturing on Matrigel, autologous human urine cells were used as feeder cells to support the survival of reprogramming cells [87]. Small molecules could provide integration-free, virus-free, and animal component-free generation of UiPSCs, which might be safe enough to establish a clinical grade UiPSCs bank for personalized medicine.

### UiNSC reprogramming

Human-derived neural stem cells (NSCs) are important for understanding the pathogenesis of neural diseases and for drug screening. However, obtaining tissues containing NSCs from patients’ brains or aborted fetus is an invasive procedure with many ethical concerns. Several models of neurological diseases using iPSCs have been established, but the differentiation process from iPSCs to NSCs is inefficient and time-consuming. Furthermore, the resulting NSCs may contain residual undifferentiated iPSCs that would lead to teratoma upon transplantation.

Lineage reprogramming could be used to generate iNSCs without passing through the iPSC state to circumvent the tumorigenic issues. It was reported that USCs could be directly reprogrammed into UiNSCs by adding a cocktail of five small molecules (CHIR99021, PD0325901, A83-01, thiazovivin, and DMH1) in the defined basal medium of USCs electroporated with episomal vectors expressing OCT4, SOX2, SV40T, KLF4, and miR-302-367 cluster [88]. The presumptive UiNSCs exhibited rosette-like morphology at day 12–15 with iPSC-like appearance at day 24–28. They could be expanded for 11 passages and remained homogeneous and expressed NSC markers SOX2 and NESTIN without OCT4 and NANOG activation. The reprogrammed UiNSCs could efficiently differentiate into β III tubulin (TUJ1)^+^ neurons and glial fibrillary acid protein (GFAP)^+^ astrocytes in vitro and in vivo. Most importantly, transplanted hiNSCs did not give rise to teratoma in rat brain. Further characterization of the individual compounds in the small molecule cocktail revealed that A83-01 alone, a selective inhibitor for TGFβ signaling, was sufficient to convert the fate of lentiviral OSKM-treated USCs into UiNSCs instead of UiPSCs [33]. In agreement with this, culturing OSKM-treated USCs in E7 medium (E8 medium without TGFβ) could easily generate NSCs, furthering indicating that removal of TGFβ is sufficient for UiNSCs formation. Consistent with the previous finding that TGFβ induces epithelial-mesenchymal transition to inhibit early phase somatic reprogramming [89], early exposure of TGFβ or overexpression of its downstream effector SNAI1 suppressed reprogramming of lentiviral OSKM-treated USCs, whereas later exposure activated the pluripotent state. These findings indicate that TGFβ activity can be manipulated at different times during USCs reprogramming to generate either iNSCs or iPSCs. Another study used a cocktail of four small molecules (A83-01, PD0325901, Thiazovivin, and CHIR99021) together with pEP4-EO2S-ET2K and pEP4-M2L plasmids containing *OCT4*, *SOX2*, *KLF4*, *SV40LT*, *c-MYC*, and *LIN28* genes to enhance the reprogramming efficiency of USCs into iPSCs and iNSCs. When using a higher concentration cocktail in the early stage of reprogramming, iPSCs appeared earlier (10 days) than iNSCs (12–15 days) [90]. This non-integrative method could generate iPSCs and iNSCs from USCs at twice the speed compared to reprogramming blood or skin cells.

Self-replicating mRNA replicon expressing OCT4, SOX2, KLF4, GLIS1, and B18R proteins together with small molecules (Purmorphamine, Forskolin, Vitamin C, and Sodium Butyrate) have also been used to generate UiNSCs [19]. These reprogrammed UiNSCs could be cultured in mild hypoxic condition, which mimics their natural niche environment to promote self-renewal capacity [91]. The UiNSCs were derived from neuroepithelial-like USCs and expressed NSC markers SOX2, NESTIN, and PAX6 within 8 days. Further treatment with purmorphamine or FGF8 to induce ventral or midbrain fate showed the majority of UiNSCs had acquired caudal identity rather than anterior character. These UiNSCs could also differentiate into functional neurons, oligodendrocytes, and astrocytes in vitro and in vivo. Furthermore, they demonstrated a lack of tumor formation upon injection into the immunodeficient nude mice. This efficient reprogramming strategy can generate UiNSCs that are transgene-free and safe for clinical applications.

## Applications of UiPSCs and UiNSCs in neurological disease modeling

Neurological diseases including spinal cord injury (SCI) and neurodegenerative disorders result from the progressive loss or damage of neurons in the central nervous system and/or the peripheral nervous system. Many diseases are caused by genetic mutations, but some diseases are complex with unknown causes. Animal models are commonly used to study disease mechanisms and to develop treatment strategies, but because of differences between animals and humans in terms of genetics, metabolism and even body size, these experimental models may not fully recapitulate disease conditions and drug responses. Establishing neurological disease models using cells from patients provides a powerful in vitro platform to study the diseases and to develop treatment strategies. Several groups have demonstrated the therapeutic potential of reprogrammed iNSCs from skin fibroblasts in neurological diseases including multiple sclerosis [95], Parkinson’s disease [96], spinal cord injury [97], and stroke [98]. However, it involves minimally invasive skin punch biopsy. Besides their non-invasive sampling, ease of reprogramming, and inherited characteristics from their original donor cells [99], USC-derived iPSCs and iNSCs can also differentiate into different neural lineages [88], making them ideal cellular sources for studying the pathogenesis of patient-specific neurodegenerative diseases. Patient-derived USCs have been successfully reprogrammed into iPSCs to study neurological diseases such as SMA [17, 93], Alzheimer’s disease [100] and spinal cord injury [76] (Table 2).

**Table 2:** Different strategies of UiPSCs/UiNSCs reprogramming and the generation of disease-specific UiPSCs

UiPSCs/UiNSCs reprogramming	Reprogramming strategies	Factors	Diseases (mutations)	Major findings	References
UiPSCs	Retrovirus	OSKM	N.A	First reported method to generate UiPSCs with reprogramming efficiency up to 4%	[32, 52]
			Paroxysmal kinesigenic dyskinesia (PKD) (Proline-rich transmembrane protein 2 (PPRT2) c.649dupC mutation)	PRRT2 mRNA was reduced in PKD-UiPSCs PKD-UiPSCs were able to differentiate into functional glutamatergic, dopaminergic, and motor neurons in vitro	[59]
			X-linked Danon disease (nonsense mutation of the *LAMP-2* gene (c.520c > T, exon 4)	Patients’ iPSC-cardiomyocytes (CMs) lines were generated Administration of the DNA demethylation agent 5-aza-2’-deoxycytidine reactivated the silent LAMP2 allele in patients’ iPSCs and iPSC-CMs and ameliorated their autophagy failure	[60]
	Lentivirus	OSKM	Systemic lupus erythematosus (SLE)	SLE patients-UiPSCs were generated	[62]
			Cryptorchid (Cryp) (mutations in *insulin-like factors 3*, *zinc finger (ZNF) 214* and *ZNF 215* genes)	Cryp-UiPSC lines were generated	[63]
			Spinal muscular atrophy (SMA) (mutations of the survival motor neuron 1 (*SMN1*) gene)	The neurite outgrowth was reduced in both SMA type I and III-UiPSCs derived motor neurons (MNs) Significant hyperexcitability was detected in SMA type I-UiPSCs derived MNs, but not in SMA type III-UiPSCs derived MNs	[17]
	Sendai Virus	OSKM	Attention deficit hyperactivity disorder (ADHD) type 2 diabetes mellitus	ADHD-UiPSCs were generated	[67]
			Obsessive compulsive disorder (OCD)	OCD-UiPSCs were generated	[68]
			Duchenne Muscular Dystrophy (DMD) (dystrophin/deletion of exon 50)	DMD-UiPSCs were generated and can be differentiated into cardiomyocytes	[69]
			DMD (DMD/*c.497G* > *T; p.G166V*)	DMD-UiPSCs were generated	[70]
			DMD (dystrophin/deletion of exon 50) (CRISPR-CAS9 generation of *c.263delG* in the *dystrophin* gene)	Reduced myofibril contractile tension, slower relaxation kinetics, and Ca^2+^ handling abnormalities	[71]
			Dilated cardiomyopathy (DCM)	DCM-UiPSCs were generated	[72]
			Heterozygous for a dinucleotide insertion within exon 4 of *PAI-1* gene	PAI-1-UiPSCs were generated	[92]
			Ventricular septal defect (VSD) (ryanodine receptor 2 (RyR2) mutation (*c.7448 T* > *G, p.L2483R*)	VSD-UiPSCs were able to differentiate into cardiomyocytes but had a higher level of autophagy	[74]
			X-linked Alport syndrome (X-LAS) (Hemizygous COL4A5 gene mutation p.G1433V (c.4298G > T)	X-LAS-UiPSCs were generated	[75]
			Spinal cord injury (SCI)	SCI UiPSCs-derived neural progenitor cells were able to give rise to neurons, oligodendrocytes, and astrocytes. Grafted neural progenitor cells into the injured spinal cord survived and differentiated into neurons and glia	[76]
		OSK, SV40, miR302-367	Spinal muscular atrophy (homozygous deletion of exon 7 and exon 8 of the *SMN1* gene)	Conversion of the SMN2 gene to an SMN1-like gene in SMA-UiPSCs using CRISPR/Cpf1 and single-stranded oligodeoxynucleotide in UiPSCs restored SMN expression and MN differentiation	[93]
		SeV, KOS, Klf4 and c-Myc	Type 2 diabetes mellitus (T2DM)	T2DM-UiPSCs differentiated into neuron, astrocyte, and microvascular endothelial cells	[77]
	Episomal vectors	OSK and SV40LT	Hemophilia A (HA)	HA-UiPSCs-derived hepatocytes failed to produced clotting factor VIII (FVIII)	[94]
		OSK, SV40T and miR-302-367	Hemophilia A, Hemophilia B, Amyotrophic lateral sclerosis (ALS), Systemic lupus erythematosus, β-thalassemia	Patients-UiPSCs were generated	[80]
		OSKM	Down syndrome (DS) (Trisomy 21-(T21)	T21-UiPSCs maintained chromosomal stability for more than 20 passages and were more sensitive to proteotoxic stress than euploid iPSCs T21-UiPSCs can be differentiated into glutamatergic neurons and cardiomyocytes	[84]
		OSK and miR-302-367	Phenylketonuria (PKU)	PKU-UiPSCs were generated	[73]
		OSKM, LIN28, NANOG and SV40LT with miR302/367	PCSK9-mediated autosomal dominant hypercholesterolemia (PCSK9-S127R (ADH) and R104C/V114A (FHBL) mutations)	PCSK9-UiPSCs differentiated into hepatocyte-like cells ADH-derived cells secreted less amount of PCSK9 with a reduction in low-density lipoprotein (LDL) uptake FHBL-derived cells showed a strong dcreased in PCSK9 secretion and an increase in LDL uptake Pravastatin treatment enhanced LDL receptor and PCSK9 mRNA expression, as well as PCSK9 secretion and LDL uptake	[43]
			Type 2 Long QT syndrome (HERG A561P mutation)	Patient-UiPSCs differentiated into CMs using the matrix sandwich method The HERG A561P mutation led to a trafficking defect with reduced delayed rectifier K^+^ current, resulting in action potential prolongation and arrhythmias	[85]
	Episomal with small molecules	L-Myc, OSK, Glis1, and miR-302 cluster with inhibitor of lysine-demethylase1, methyl ethyl ketone, glycogen synthase kinase 3β, and histone deacetylase	N.A	Decreased chromosomal variation and increased Sir1 expression in UiPSCs compared with iPSCs induced using the traditional episomal system	[86]
	Small molecules	cyclic pifithrin-α (a P53 inhibitor), A-83-01, CHIR99021, thiazovivin, NaB, and PD0325901	Diabetes and blood disorders	Improved the reprogramming efficiency (170-foldmore) significantly Replacement of Matrigel with autologous urine cell feeders can overcome the reprogramming failure	[87]
UiNSCs reprogramming	Small molecules with episomal vectors	CHIR99021, PD0325901, A83-01, thiazovivin, and DMH1 with OSK, SV40T, and miR-302-367 cluster	N.A	The UiNSCs can self-renew and differentiate into multiple functional neuronal subtypes and glial cells in vitro	[88]
		CHIR99021, PD0325901, A83-01, Thiazovivin with *OSK*, *SV40LT*, *c-MYC*, and *LIN28*	N.A	The UiNSCs generated were positive for NSC markers NESTIN, PAX6, SOX2, and OLIG2	[90]
	mRNA with small molecules	OSK, GLIS1 and B18R mRNAs with purmorphamine, Forskolin,Vitamin C, and Sodium Butyrate	N.A	The UiNSCs generated can differentiate into neurons, astrocytes and oligodendrocytes in vitro and in vivo	[19]

### Spinal muscular atrophy

Spinal muscular atrophy is a rare autosomal recessive disorder causing infant mortality, and has an incidence of 1 in 10,000 live births and a carrier frequency of about 1 in 50 [101]. A homozygous deletion or mutation of the survival of motor neurons 1 (*SMN1*) gene [102, 103] in SMA causes deficient full-length SMN protein, leading to degeneration of spinal motor neurons, denervation of skeletal muscle, muscular atrophy, and eventually death [104]. The closely related *SMN2* gene predominantly encodes a truncated protein due to alternative splicing, and produces an SMN protein with 10%-20% functionality, which can compensate for the lack of *SMN1* gene in SMA patients depending on the *SMN2* copy number [105]. The copy number of *SMN2* gene is inversely correlated with the severity of SMA symptoms, ranging from severe type I SMA (1–2 copies of *SMN2*) to mild type IV SMA (4–6 copies of *SMN2*) [106]. A previous study used type I (2 copies of *SMN2*) and type III (3 copies of *SMN2*) cells from the urine of SMA patients to generate UiPSCs by lentiviral-OSKM to investigate SMA in vitro [17]. Consistent with the *SMN2* copy number, UiPSCs derived from type I cells exhibited the lowest amount of SMN proteins compared to type III cells and healthy controls, and these differences carried through into the MN populations. Although there were no significant differences among the three groups in terms of the number of OLIG2^+^ spinal MN progenitors (MNPs), differentiating MNs (HB9^+^/ISL1^+^), and mature MNs (ChAT^+^), the neurite outgrowth from type I and III SMA clones were significantly reduced compared to the control. The SMN protein is involved in the assembly of U12 spliceosome that contributes to the alternative splicing, hence, SMN deficiency leads to disrupted U12 splicing and mRNA expression required for motor circuit function [107]. Alternatively, low SMN expression resulted in the activation of Rho/ROCK and JNK signaling pathways, which may mediate the neuronal growth dysfunction in SMA [108]. In addition, type I SMA MNs, but not type III, displayed hyperexcitability with enhanced Na^+^ channel activities. Whether this abnormal neuronal firing is a cell autonomous event remains unknown [109, 110]. Nevertheless, modeling SMA disease using patient urine cells provides a simple and non-invasive strategy to reveal the molecular mechanisms underlying MN defects in SMA.

On the other hand, organoid spinal cord differentiated from skin fibroblast-derived iPSCs from SMA patients could also recapitulate the neurological conditions of SMA [111]. The MNs in SMA spinal organoids had upregulated cell cycle genes (*CDK1*, *CDK2*, *CCNA2*, *CCNB1,* and *CCNB2*) and could subsequently re-enter the cell cycle leading to MN death. Treatment with CDK inhibitor prolonged the survival of SMA MNs. Besides, another report [93] used CRISPR/Cpf1 and single-stranded oligodeoxynucleotide to edit *SMN2* gene to an SMN1-like gene in SMA UiPSCs generated by an episomal reprogramming vector. Restoration of the SMN expression rescued MN generation. This study provides proof-of-principle for establishing a gene correction approach for the treatment of SMA.

### Spinal cord injury

Traumatic injury to the spinal cord can result in severed axons and neuronal death, leading to motor and sensory dysfunction. Currently, there are no effective treatments for spinal cord injury. Transplantation of NSCs has shown promise as a treatment for spinal cord injury. Although transplantation of hNSCs (derived from fetal brain, spinal cord, or hESCs) into a spinal cord-injury mice model demonstrated promising results of locomotor recovery [112–114], these cell sources have raised ethical concerns. In addition, the derived cells are allogeneic and require life-long immunosuppression to suppress immune rejection. A previous study generated iPSCs from patients’ urine by Sendai virus carrying OSKM, and further differentiated them into NSCs using appropriate neural induction medium [76]. These UiNSCs were grafted into spinal cord-injury mice giving rise to neurons and astrocytes in the injured environment without tumor formation, although locomotor recovery in these grafted mice was not examined. Nevertheless, this study demonstrates the therapeutic potential of UiNSCs which are transgene-free and safe for the transplantation treatment of spinal cord injury.

Although similar non-integrated reprogramming strategy was used to generate pluripotent UiPSCs from an Alzheimer’s disease patient’s urine, no further characterization of their ability to differentiate into neural lineages in vitro and in animal models were conducted [100].

## Direct reprogramming of USCs into NSCs or neurons

Several studies have demonstrated that USCs can be reprogrammed into neurons without going through the stage of iPSC or iNSC generation. This can be accomplished by different approaches including retroviral-driven expression of transcription factors, matrix and small molecules. The iNSCs can differentiate into different neuronal subtypes and glial cells in vitro. Apart from expressing characteristic markers, the induced neurons (iNs) showed extensive neurite outgrowth and generated action potential, indicating the ability to generate functional neurons in vitro by direct reprogramming of USCs. A recent review has provided a detailed summary of different direct reprogramming strategies in generating iNSCs or iNs from USCs [115]. Several of these studies used small molecules as a non-integration approach which is faster than going through iPSC to generate neurons or NSCs from USCs [38, 91, 116], providing a more effective and safer strategy for modeling neurological diseases and developing therapies respectively.

## Current challenges and Future perspectives

Despite aforementioned advantageous of using USCs to generate target cell types for modeling CNS defects in patient-specific manner, there are still several issues yet to be resolved for further basic research and clinical applications. Firstly, microorganism contamination in urine remains an issue of USCs sampling compared to other somatic cells. Addition of normocure, a broad-spectrum antibacterial agent that can eliminate microorganism in unsterile floor-collected urine samples without affecting the growth of USCs in culture [117]. Secondly, it is a double-edged sword that UiPSCs from individuals carrying specific genetic background may impact the differentiation propensities into a specific lineage under the same culture condition that makes comparison difficult, but the distinct genetic differences give us clues about the specific determinants of disease severity and the response to drug treatment on an individual basis that provides an important ground for future personalized medicine. Ideally, the USCs from patients with genetic diseases for disease modeling should be compared with the USCs from their healthy relatives or siblings with similar genetic background. However, this is not always practically feasible. To resolve the impact of genetic differences between individual on disease modeling, genome editing technology such as the CRISPR/Cas9 system could be used to create disease mutation or deletion in UiPSCs and the healthy control UiPSCs originated from the same individual, enabling us to reveal the disease mechanism caused by a specific genetic defect [118, 119]. A recent study using CRISPR/Cas9-mediated beta-globin gene correlation of sickle cell disease patient-derived hematopoietic stem cells in combination with autologous transplantation underlies its potential application in generating gene-correlated autologous UiPSCs-derived cell type for the treatment of genetic diseases [120]. Thirdly, it has been reported that iPSCs from different somatic origins harbor different patterns of epigenetic signatures, which bias their differentiation potency to specific lineages related to the donor cell while antagonizing other cell fates. Such an “epigenetic memory” of the donor tissue could compromise the iPS reprogramming efficiency and the differentiation propensity of iPSCs to generate target cell type for disease modeling and treatment [121]. This issue can be overcome by adding vitamin C [122] or histone deacetylase inhibitor valproic acid (VPA) [123] in the culture medium for proper DNA demethylation and histone acetylation in somatic cell genome to enhance reprogramming efficiency of USCs into iPSCs [32] and neurons [50]. Last but not least, the aforementioned studies investigated patient-specific UiPSCs and their target cell types in 2D culture which does not mimic the disease conditions in vivo. Advances in organoid technology are anticipated to bridge the gap between two-dimensional cell models and three-dimensional in vivo models. Recent studies have successfully generated brain and spinal organoids, which can virtually recapitulate the development of the CNS and diseases [124, 125]. Martins et al. demonstrated the ability to generate common progenitor cells for posterior spinal cord and muscle, allowing the formation of functional neuromuscular junctions in single organoids [125]. This study has opened new avenues of research for modeling neuromuscular defects in three-dimensional neural tissues generated from UiPSCs of SMA patients. In addition, SMA organoids can be used to evaluate current treatment efficacy, toxicity and pharmacokinetics, as well as optimize personal treatment strategies for patients.

## Conclusion

**Fig. 2:**
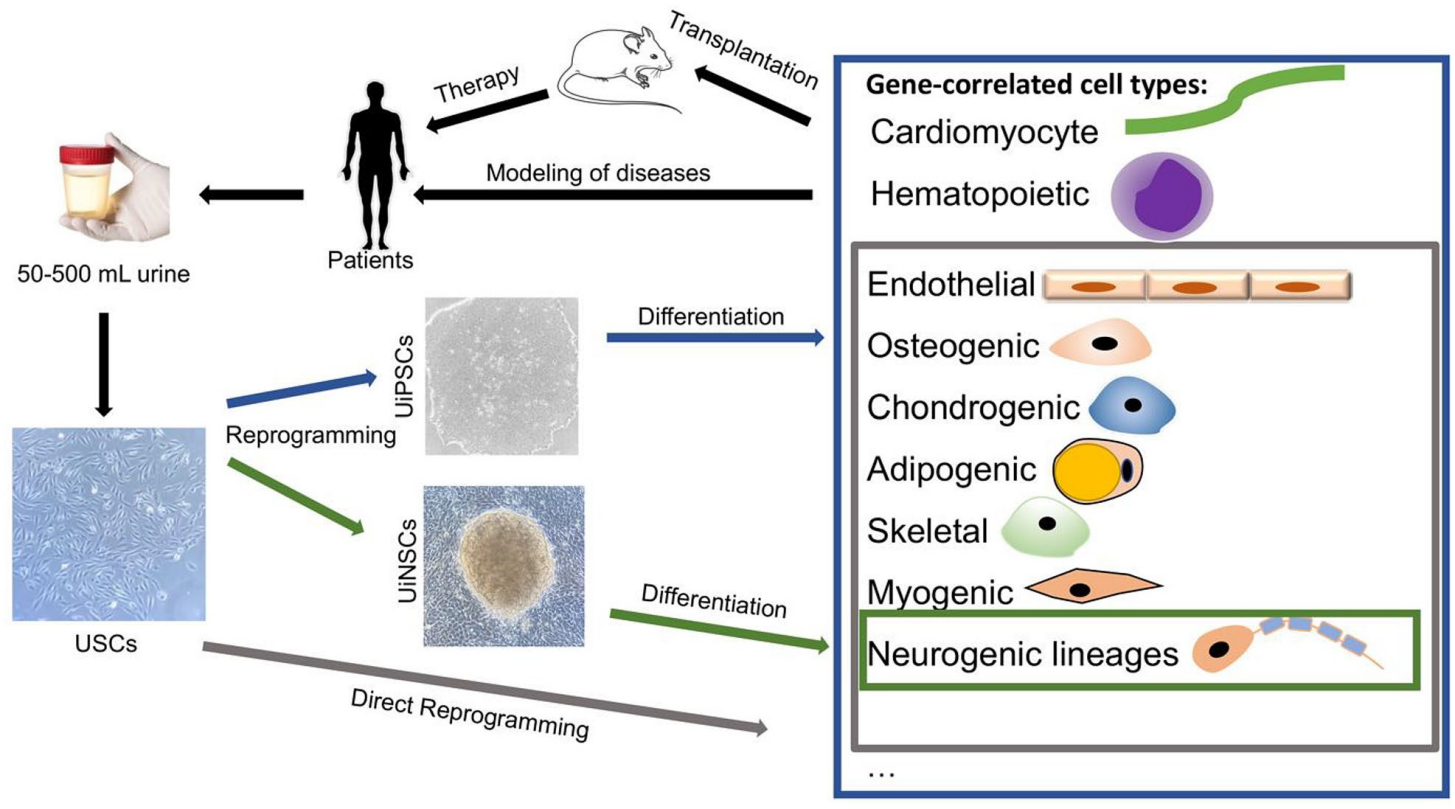
Schematic diagram showing the broad applications of USCs as cellular models of human diseases. Patients-specific USCs can be collected by non-invasive methods for reprogramming into UiPSCs, UiNSCs or different cell types for disease modeling and functional assessment of gene-corrected cell types by transplantation into rodent model before applying to human, paving the way for personalized medicine

USCs serve as a useful non-invasive cell source for disease modeling with high proliferation and differentiation abilities, and the development of UiPSC/UiNSC-based technology holds enormous potential for clinical applications in personalized medicine (Fig. 2). For long-term therapeutic potential, efficacy and safety of gene-edited UiNSCs or neural organoids need to be critically evaluated in pre-clinical rodent models and in large animal models that more closely mimic the neuropathological features of humans.

## Data Availability

Data will be provided upon request.

## References

[1] Velasco S, Ibrahim MM, Kakumanu A, Garipler G, Aydin B, Al-Sayegh MA, Hirsekorn A, Abdul-Rahman F, Satija R, Ohler U, Mahony S, Mazzoni EO (2017). A multi-step transcriptional and chromatin state cascade underlies motor neuron programming from embryonic stem cells. Cell Stem Cell.

[2] Noisa P, Raivio T, Cui W (2015). Neural progenitor cells derived from human embryonic stem cells as an origin of dopaminergic neurons. Stem Cells Int..

[3] Chung SH, Shen W, Davidson KC, Pebay A, Wong RCB, Yau B, Gillies M (2019). Differentiation of retinal glial cells from human embryonic stem cells by promoting the notch signaling pathway. Front Cell Neurosci.

[4] Byun JS, Lee CO, Oh M, Cha D, Kim W-K, Oh K-J, Bae K-H, Lee S-C, Han B-S (2020). Rapid differentiation of astrocytes from human embryonic stem cells. Neurosci Lett..

[5] Sumitha R, Manjunatha VM, Sabitha RK, Alladi PA, Nalini A, Rao LT, Chandrasekhar Sagar BK, Steinbusch HWM, Kramer BW, Sathyaprabha TN, Raju TR (2019). Cerebrospinal fluid from patients with sporadic amyotrophic lateral sclerosis induces degeneration of motor neurons derived from human embryonic stem cells. Mol Neurobiol.

[6] Birger A, Ottolenghi M, Perez L, Reubinoff B, Behar O (2018). ALS-related human cortical and motor neurons survival is differentially affected by Sema3A. Cell Death Dis.

[7] Hall CE, Yao Z, Choi M, Tyzack GE, Serio A, Luisier R, Harley J, Preza E, Arber C, Crisp SJ, Watson PMD, Kullmann DM, Abramov AY, Wray S, Burley R, Loh SHY, Martins LM, Stevens MM, Luscombe NM, Sibley CR, Lakatos A, Ule J, Gandhi S, Patani R (2017). Progressive motor neuron pathology and the role of astrocytes in a human stem cell model of VCP-related ALS. Cell Rep.

[8] Xu C-C, Denton KR, Wang Z-B, Zhang X, Li X-J (2016). Abnormal mitochondrial transport and morphology as early pathological changes in human models of spinal muscular atrophy. Dis Model Mech.

[9] Parker GC, Carruthers NJ, Gratsch T, Caruso JA, Stemmer PM (2017). Proteomic profile of embryonic stem cells with low survival motor neuron protein is consistent with developmental dysfunction. J Neural Transm (Vienna).

[10] Takahashi K, Yamanaka S (2006). Induction of pluripotent stem cells from mouse embryonic and adult fibroblast cultures by defined factors. Cell.

[11] Nekrasov ED, Vigont VA, Klyushnikov SA, Lebedeva OS, Vassina EM, Bogomazova AN, Chestkov IV, Semashko TA, Kiseleva E, Suldina LA, Bobrovsky PA, Zimina OA, Ryazantseva MA, Skopin AY, Illarioshkin SN, Kaznacheyeva EV, Lagarkova MA, Kiselev SL (2016). Manifestation of Huntington's disease pathology in human induced pluripotent stem cell-derived neurons. Mol Neurodegener.

[12] Yagi T, Ito D, Okada Y, Akamatsu W, Nihei Y, Yoshizaki T, Yamanaka S, Okano H, Suzuki N (2011). Modeling familial Alzheimer's disease with induced pluripotent stem cells. Hum Mol Genet.

[13] Mungenast AE, Siegert S, Tsai LH (2016). Modeling Alzheimer's disease with human induced pluripotent stem (iPS) cells. Mol Cell Neurosci.

[14] Osaki T, Uzel SGM, Kamm RD (2018). Microphysiological 3D model of amyotrophic lateral sclerosis (ALS) from human iPS-derived muscle cells and optogenetic motor neurons. Sci Adv..

[15] Alves CJ, Dariolli R, Jorge FM, Monteiro MR, Maximino JR, Martins RS, Strauss BE, Krieger JE, Callegaro D, Chadi G (2015). Gene expression profiling for human iPS-derived motor neurons from sporadic ALS patients reveals a strong association between mitochondrial functions and neurodegeneration. Front Cell Neurosci.

[16] Fuller HR, Mandefro B, Shirran SL, Gross AR, Kaus AS, Botting CH, Morris GE, Sareen D (2015). Spinal muscular atrophy patient iPSC-derived motor neurons have reduced expression of proteins important in neuronal development. Front Cell Neurosci.

[17] Lin X, Li J-J, Qian W-J, Zhang Q-J, Wang Z-F, Lu Y-Q, Dong E-L, He J, Wang N, Ma L-X, Chen W-J (2017). Modeling the differential phenotypes of spinal muscular atrophy with high-yield generation of motor neurons from human induced pluripotent stem cells. Oncotarget.

[18] Ng S-Y, Soh Boon S, Rodriguez-Muela N, Hendrickson David G, Price F, Rinn John L, Rubin LL (2015). Genome-wide RNA-Seq of human motor neurons implicates selective er stress activation in spinal muscular atrophy. Cell Stem Cell.

[19] Kang PJ, Son D, Ko TH, Hong W, Yun W, Jang J, Choi JI, Song G, Lee J, Kim IY, You S (2019). mRNA-driven generation of transgene-free neural stem cells from human urine-derived cells. Cells.

[20] Xiao D, Liu X, Zhang M, Zou M, Deng Q, Sun D, Bian X, Cai Y, Guo Y, Liu S, Li S, Shiang E, Zhong H, Cheng L, Xu H, Jin K, Xiang M (2018). Direct reprogramming of fibroblasts into neural stem cells by single non-neural progenitor transcription factor Ptf1a. Nat Commun.

[21] Zhang M, Lin YH, Sun YJ, Zhu S, Zheng J, Liu K, Cao N, Li K, Huang Y, Ding S (2016). Pharmacological reprogramming of fibroblasts into neural stem cells by signaling-directed transcriptional activation. Cell Stem Cell.

[22] Lowry WE, Richter L, Yachechko R, Pyle AD, Tchieu J, Sridharan R, Clark AT, Plath K (2008). Generation of human induced pluripotent stem cells from dermal fibroblasts. Proc Natl Acad Sci U S A.

[23] Loh YH, Agarwal S, Park IH, Urbach A, Huo H, Heffner GC, Kim K, Miller JD, Ng K, Daley GQ (2009). Generation of induced pluripotent stem cells from human blood. Blood.

[24] Fernandes IR, Russo FB, Pignatari GC, Evangelinellis MM, Tavolari S, Muotri AR, Beltrao-Braga PC (2016). Fibroblast sources: Where can we get them?. Cytotechnology.

[25] Bharadwaj S, Liu G, Shi Y, Markert C, Andersson KE, Atala A, Zhang Y (2011). Characterization of urine-derived stem cells obtained from upper urinary tract for use in cell-based urological tissue engineering. Tissue Eng Part A.

[26] Wang B, Hu Y, Liu L, Hu K, Tie R, He Y, Fu S, Zhu N, Luo Y, Yu X, Huang H (2015). Phenotypical and functional characterization of bone marrow mesenchymal stem cells in patients with chronic graft-versus-host disease. Biol Blood Marrow Transplant.

[27] Mehrabani D, Nazarabadi RB, Kasraeian M, Tamadon A, Dianatpour M, Vahdati A, Zare S, Ghobadi F (2016). Growth kinetics, characterization, and plasticity of human menstrual blood stem cells. Iran J Med Sci.

[28] Wu C, Chen L, Huang YZ, Huang Y, Parolini O, Zhong Q, Tian X, Deng L (2018). Comparison of the proliferation and differentiation potential of human urine-, placenta decidua basalis-, and bone marrow-derived stem cells. Stem Cells Int.

[29] Mack AA, Kroboth S, Rajesh D, Wang WB (2011). Generation of induced pluripotent stem cells from CD34+ cells across blood drawn from multiple donors with non-integrating episomal vectors. PLoS ONE.

[30] Chen A-J, Pi J-K, Hu J-G, Huang Y-Z, Gao H-W, Li S-F, Li-Ling J, Xie H-Q (2019). Identification and characterization of two morphologically distinct stem cell subpopulations from human urine samples. Sci China Life Sci..

[31] Gao P, Han P, Jiang D, Yang S, Cui Q, Li Z (2017). Effects of the donor age on proliferation, senescence and osteogenic capacity of human urine-derived stem cells. Cytotechnology.

[32] Zhou T, Benda C, Dunzinger S, Huang Y, Ho JC, Yang J, Wang Y, Zhang Y, Zhuang Q, Li Y, Bao X, Tse H-F, Grillari J, Grillari-Voglauer R, Pei D, Esteban MA (2012). Generation of human induced pluripotent stem cells from urine samples. Nat Protoc.

[33] Wang L, Li X, Huang W, Zhou T, Wang H, Lin A, Hutchins AP, Su Z, Chen Q, Pei D, Pan G (2016). TGFbeta signaling regulates the choice between pluripotent and neural fates during reprogramming of human urine derived cells. Sci Rep.

[34] Sutherland GR, Bain AD (1972). Culture of cells from the urine of newborn children. Nature.

[35] Zhang Y, McNeill E, Tian H, Soker S, Andersson KE, Yoo JJ, Atala A (2008). Urine derived cells are a potential source for urological tissue reconstruction. J Urol.

[36] Schosserer M, Reynoso R, Wally V, Jug B, Kantner V, Weilner S, Buric I, Grillari J, Bauer JW, Grillari-Voglauer R (2015). Urine is a novel source of autologous mesenchymal stem cells for patients with epidermolysis bullosa. BMC Res Notes.

[37] Falzarano MS, Ferlini A (2019). Urinary stem cells as tools to study genetic disease: overview of the literature. J Clin Med..

[38] Guan JJ, Niu X, Gong FX, Hu B, Guo SC, Lou YL, Zhang CQ, Deng ZF, Wang Y (2014). Biological characteristics of human-urine-derived stem cells: potential for cell-based therapy in neurology. Tissue Eng Part A.

[39] Dorrenhaus A, Muller JI, Golka K, Jedrusik P, Schulze H, Follmann W (2000). Cultures of exfoliated epithelial cells from different locations of the human urinary tract and the renal tubular system. Arch Toxicol.

[40] Tayhan SE, Keles GT, Topcu I, Mir E, Gurhan SID (2017). Isolation and in vitro cultivation of human urine-derived cells: an alternative stem cell source. Turk J Urol.

[41] Chen AJ, Pi JK, Hu JG, Huang YZ, Gao HW, Li SF, Li-Ling J, Xie HQ (2020). Identification and characterization of two morphologically distinct stem cell subpopulations from human urine samples. Sci China Life Sci.

[42] Bharadwaj S, Liu G, Shi Y, Wu R, Yang B, He T, Fan Y, Lu X, Zhou X, Liu H, Atala A, Rohozinski J, Zhang Y (2013). Multipotential differentiation of human urine-derived stem cells: potential for therapeutic applications in urology. Stem Cells.

[43] Si-Tayeb K, Idriss S, Champon B, Caillaud A, Pichelin M, Arnaud L, Lemarchand P, Le May C, Zibara K, Cariou B (2016). Urine-sample-derived human induced pluripotent stem cells as a model to study PCSK9-mediated autosomal dominant hypercholesterolemia. Dis Model Mech.

[44] He W, Zhu W, Cao Q, Shen Y, Zhou Q, Yu P, Liu X, Ma J, Li Y, Hong K (2016). Generation of mesenchymal-like stem cells from urine in pediatric patients. Transplant Proc.

[45] Van der Hauwaert C, Savary G, Gnemmi V, Glowacki F, Pottier N, Bouillez A, Maboudou P, Zini L, Leroy X, Cauffiez C, Perrais M, Aubert S (2013). Isolation and characterization of a primary proximal tubular epithelial cell model from human kidney by CD10/CD13 double labeling. PLoS ONE.

[46] Zhang SZ, Ma LX, Qian WJ, Li HF, Wang ZF, Wang HX, Wu ZY (2016). Modeling neurological disease by rapid conversion of human urine cells into functional neurons. Stem Cells Int.

[47] Guan J, Zhang J, Guo S, Zhu H, Zhu Z, Li H, Wang Y, Zhang C, Chang J (2015). Human urine-derived stem cells can be induced into osteogenic lineage by silicate bioceramics via activation of the Wnt/beta-catenin signaling pathway. Biomaterials.

[48] Chen L, Li L, Xing F, Peng J, Peng K, Wang Y, Xiang Z (2018). Human urine-derived stem cells: potential for cell-based therapy of cartilage defects. Stem Cells Int.

[49] Liu D, Rychkov G, Al-Hawwas M, Manaph NPA, Zhou F, Bobrovskaya L, Liao H, Zhou X-F (2020). Conversion of human urine-derived cells into neuron-like cells by small molecules. Mol Biol Rep..

[50] Xu G, Wu F, Gu X, Zhang J, You K, Chen Y, Getachew A, Zhuang Y, Zhong X, Lin Z, Guo D, Yang F, Pan T, Wei H, Li YX (2019). Direct conversion of human urine cells to neurons by small molecules. Sci Rep.

[51] Kang HS, Choi SH, Kim BS, Choi JY, Park GB, Kwon TG, Chun SY (2015). Advanced properties of urine derived stem cells compared to adipose tissue derived stem cells in terms of cell proliferation, immune modulation and multi differentiation. J Korean Med Sci.

[52] Zhou T, Benda C, Duzinger S, Huang Y, Li X, Li Y, Guo X, Cao G, Chen S, Hao L, Chan Y-C, Ng K-M, Ho JC, Wieser M, Wu J, Redl H, Tse H-F, Grillari J, Grillari-Voglauer R, Pei D, Esteban MA (2011). Generation of induced pluripotent stem cells from urine. J Am Soc Nephrol.

[53] Zafarullah M, Jasoliya M, Tassone F (2020). Urine-derived epithelial cell lines: a new tool to model fragile X syndrome (FXS). Cells.

[54] Choi JY, Chun SY, Ha Y-S, Kim DH, Kim J, Song PH, Kim HT, Yoo ES, Kim BS, Kwon TG (2017). Potency of human urine-derived stem cells for renal lineage differentiation. Tissue Eng Regen Med.

[55] Kim JY, Chun SY, Park J-S, Chung J-W, Ha Y-S, Lee JN, Kwon TG (2018). Laminin and platelet-derived growth factor-BB promote neuronal differentiation of human urine-derived stem cells. Tissue Eng Regen Med.

[56] Zidan AA, Perkins GB, Al-Hawwas M, Elhossiny A, Yang J, Bobrovskaya L, Mourad GM, Zhou XF, Hurtado PR (2021). Urine stem cells are equipped to provide B cell survival signals. Stem Cells..

[57] Duan YR, Chen BP, Chen F, Yang SX, Zhu CY, Ma YL, Li Y, Shi J (2019). Exosomal microRNA-16–5p from human urine-derived stem cells ameliorates diabetic nephropathy through protection of podocyte. J Cell Mol Med..

[58] Rao MS, Malik N (2012). Assessing iPSC reprogramming methods for their suitability in translational medicine. J Cell Biochem.

[59] Zhang SZ, Li HF, Ma LX, Qian WJ, Wang ZF, Wu ZY (2015). Urine-derived induced pluripotent stem cells as a modeling tool for paroxysmal kinesigenic dyskinesia. Biol Open.

[60] Ng KM, Mok PY, Butler AW, Ho JC, Choi SW, Lee YK, Lai WH, Au KW, Lau YM, Wong LY, Esteban MA, Siu CW, Sham PC, Colman A, Tse HF (2016). Amelioration of X-linked related autophagy failure in danon disease with DNA methylation inhibitor. Circulation.

[61] Jiang YF, Chen M, Zhang NN, Yang HJ, Rui Q, Zhou YF (2018). In vitro and in vivo differentiation of induced pluripotent stem cells generated from urine-derived cells into cardiomyocytes. Biol Open..

[62] Chen Y, Luo R, Xu Y, Cai X, Li W, Tan K, Huang J, Dai Y (2013). Generation of systemic lupus erythematosus-specific induced pluripotent stem cells from urine. Rheumatol Int.

[63] Zhou J, Wang X, Zhang S, Gu Y, Yu L, Wu J, Gao T, Chen F (2013). Generation and characterization of human cryptorchid-specific induced pluripotent stem cells from urine. Stem Cells Dev.

[64] Kane NM, Nowrouzi A, Mukherjee S, Blundell MP, Greig JA, Lee WK, Houslay MD, Milligan G, Mountford JC, von Kalle C, Schmidt M, Thrasher AJ, Baker AH (2010). Lentivirus-mediated reprogramming of somatic cells in the absence of transgenic transcription factors. Mol Ther.

[65] Baum C (2007). Insertional mutagenesis in gene therapy and stem cell biology. Curr Opin Hematol.

[66] Kustikova O, Fehse B, Modlich U, Yang M, Dullmann J, Kamino K, von Neuhoff N, Schlegelberger B, Li Z, Baum C (2005). Clonal dominance of hematopoietic stem cells triggered by retroviral gene marking. Science.

[67] Sochacki J, Devalle S, Reis M, Mattos P, Rehen S (2016). Generation of urine iPS cell lines from patients with Attention Deficit Hyperactivity Disorder (ADHD) using a non-integrative method. Stem Cell Res.

[68] Sochacki J, Devalle S, Reis M, Fontenelle LF, Rehen S (2016). Generation of urine iPS cell line from a patient with obsessive-compulsive disorder using a non-integrative method. Stem Cell Res.

[69] Afzal MZ, Strande JL (2015). Generation of induced pluripotent stem cells from muscular dystrophy patients: efficient integration-free reprogramming of urine derived cells. J Vis Exp.

[70] Su J, Wang J, Wang L, Li T, Wang H, Shen J, Wang H, Zhang J, Lin W, Huang J, Liang P (2020). Generation of five induced pluripotent stem cell lines with DMD/c.497G > T mutation from renal epithelial cells of a Duchenne muscular dystrophy patient and a recessive carrier parent. Stem Cell Res..

[71] Pioner JM, Guan X, Klaiman JM, Racca AW, Pabon L, Muskheli V, Macadangdang J, Ferrantini C, Hoopmann MR, Moritz RL, Kim DH, Tesi C, Poggesi C, Murry CE, Childers MK, Mack DL, Regnier M (2020). Absence of full-length dystrophin impairs normal maturation and contraction of cardiomyocytes derived from human-induced pluripotent stem cells. Cardiovasc Res.

[72] Lin YH, Chen XM, Zhang JW, He XQ, Dai WJ, Chen MS (2016). Preclinical study on induction of pluripotent stem cells from urine of dilated cardiomyopathy patients. Eur Rev Med Pharmacol Sci.

[73] Qi Z, Cui Y, Shi L, Luan J, Zhou X, Han J (2018). Generation of urine-derived induced pluripotent stem cells from a patient with phenylketonuria. Intractable Rare Dis Res.

[74] Cao Y, Xu J, Wen J, Ma X, Liu F, Li Y, Chen W, Sun L, Wu Y, Li S, Li J, Huang G (2018). Generation of a urine-derived Ips cell line from a patient with a ventricular septal defect and heart failure and the robust differentiation of these cells to cardiomyocytes via small molecules. Cell Physiol Biochem.

[75] Guo X, Ji W, Niu C, Ding Y, Chen Z, Chen C, Tong H, Han Z, Chu M (2020). Generation of an urine-derived induced pluripotent stem cell line from a 5-year old X-linked Alport syndrome (X-LAS) patient. Stem Cell Res..

[76] Liu Y, Zheng Y, Li S, Xue H, Schmitt K, Hergenroeder GW, Wu J, Zhang Y, Kim DH, Cao Q (2017). Human neural progenitors derived from integration-free iPSCs for SCI therapy. Stem Cell Res.

[77] Liu W, Zhang P, Tan J, Lin Y (2020). Differentiation of urine-derived induced pluripotent stem cells to neurons, astrocytes, and microvascular endothelial cells from a diabetic patient. Cell Reprogram.

[78] Uhm KO, Jo EH, Go GY, Kim SJ, Choi HY, Im YS, Ha HY, Jung JW, Koo SK (2017). Generation of human induced pluripotent stem cells from urinary cells of a healthy donor using a non-integration system. Stem Cell Res.

[79] Fusaki N, Ban H, Nishiyama A, Saeki K, Hasegawa M (2009). Efficient induction of transgene-free human pluripotent stem cells using a vector based on Sendai virus, an RNA virus that does not integrate into the host genome. Proc Jpn Acad Ser B Phys Biol Sci.

[80] Xue Y, Cai X, Wang L, Liao B, Zhang H, Shan Y, Chen Q, Zhou T, Li X, Hou J, Chen S, Luo R, Qin D, Pei D, Pan G (2013). Generating a non-integrating human induced pluripotent stem cell bank from urine-derived cells. PLoS ONE.

[81] Judson RL, Babiarz JE, Venere M, Blelloch R (2009). Embryonic stem cell-specific microRNAs promote induced pluripotency. Nat Biotechnol.

[82] Lin SL, Chang DC, Ying SY, Leu D, Wu DT (2010). MicroRNA miR-302 inhibits the tumorigenecity of human pluripotent stem cells by coordinate suppression of the CDK2 and CDK4/6 cell cycle pathways. Cancer Res.

[83] Samavarchi-Tehrani P, Golipour A, David L, Sung HK, Beyer TA, Datti A, Woltjen K, Nagy A, Wrana JL (2010). Functional genomics reveals a BMP-driven mesenchymal-to-epithelial transition in the initiation of somatic cell reprogramming. Cell Stem Cell.

[84] Lee YM, Zampieri BL, Scott-McKean JJ, Johnson MW, Costa ACS (2017). Generation of integration-free induced pluripotent stem cells from urine-derived cells isolated from individuals with down syndrome. Stem Cells Transl Med..

[85] Jouni M, Si-Tayeb K, Es-Salah-Lamoureux Z, Latypova X, Champon B, Caillaud A, Rungoat A, Charpentier F, Loussouarn G, Baro I, Zibara K, Lemarchand P, Gaborit N (2015). Toward personalized medicine: using cardiomyocytes differentiated from urine-derived pluripotent stem cells to recapitulate electrophysiological characteristics of type 2 long QT syndrome. J Am Heart Assoc..

[86] Wang L, Chen Y, Guan C, Zhao Z, Li Q, Yang J, Mo J, Wang B, Wu W, Yang X, Song L, Li J (2017). Using low-risk factors to generate non-integrated human induced pluripotent stem cells from urine-derived cells. Stem Cell Res Ther.

[87] Li D, Wang L, Hou J, Shen Q, Chen Q, Wang X, Du J, Cai X, Shan Y, Zhang T, Zhou T, Shi X, Li Y, Zhang H, Pan G (2016). Optimized approaches for generation of integration-free ipscs from human urine-derived cells with small molecules and autologous feeder. Stem Cell Reports.

[88] Wang L, Wang L, Huang W, Su H, Xue Y, Su Z, Liao B, Wang H, Bao X, Qin D, He J, Wu W, So KF, Pan G, Pei D (2013). Generation of integration-free neural progenitor cells from cells in human urine. Nat Methods.

[89] Li R, Liang J, Ni S, Zhou T, Qing X, Li H, He W, Chen J, Li F, Zhuang Q, Qin B, Xu J, Li W, Yang J, Gan Y, Qin D, Feng S, Song H, Yang D, Zhang B, Zeng L, Lai L, Esteban MA, Pei D (2010). A mesenchymal-to-epithelial transition initiates and is required for the nuclear reprogramming of mouse fibroblasts. Cell Stem Cell.

[90] Cheng L, Lei Q, Yin C, Wang HY, Jin K, Xiang M (2017). Generation of urine cell-derived non-integrative human iPSCs and iNSCs: a step-by-step optimized protocol. Front Mol Neurosci.

[91] Cheng L, Hu W, Qiu B, Zhao J, Yu Y, Guan W, Wang M, Yang W, Pei G (2014). Generation of neural progenitor cells by chemical cocktails and hypoxia. Cell Res.

[92] Afzal MZ, Gartz M, Klyachko EA, Khan SS, Shah SJ, Gupta S, Shapiro AD, Vaughan DE, Strande JL (2017). Generation of human iPSCs from urine derived cells of patient with a novel heterozygous PAI-1 mutation. Stem Cell Res.

[93] Zhou M, Hu Z, Qiu L, Zhou T, Feng M, Hu Q, Zeng B, Li Z, Sun Q, Wu Y, Liu X, Wu L, Liang D (2018). Seamless genetic conversion of SMN2 to SMN1 via CRISPR/Cpf1 and single-stranded oligodeoxynucleotides in spinal muscular atrophy patient-specific induced pluripotent stem cells. Hum Gene Ther.

[94] Jia B, Chen S, Zhao Z, Liu P, Cai J, Qin D, Du J, Wu C, Chen Q, Cai X, Zhang H, Yu Y, Pei D, Zhong M, Pan G (2014). Modeling of hemophilia A using patient-specific induced pluripotent stem cells derived from urine cells. Life Sci.

[95] Sullivan GM, Knutsen AK, Peruzzotti-Jametti L, Korotcov A, Bosomtwi A, Dardzinski BJ, Bernstock JD, Rizzi S, Edenhofer F, Pluchino S, Armstrong RC (2020). Transplantation of induced neural stem cells (iNSCs) into chronically demyelinated corpus callosum ameliorates motor deficits. Acta Neuropathol Commun.

[96] Choi DH, Kim JH, Kim SM, Kang K, Han DW, Lee J (2017). Therapeutic potential of induced neural stem cells for parkinson's disease. Int J Mol Sci..

[97] Hong JY, Lee SH, Lee SC, Kim JW, Kim KP, Kim SM, Tapia N, Lim KT, Kim J, Ahn HS, Ko K, Shin CY, Lee HT, Scholer HR, Hyun JK, Han DW (2014). Therapeutic potential of induced neural stem cells for spinal cord injury. J Biol Chem.

[98] Yamashita T, Liu W, Matsumura Y, Miyagi R, Zhai Y, Kusaki M, Hishikawa N, Ohta Y, Kim SM, Kwak TH, Han DW, Abe K (2017). Novel therapeutic transplantation of induced neural stem cells for stroke. Cell Transplant.

[99] Guan X, Mack DL, Moreno CM, Strande JL, Mathieu J, Shi Y, Markert CD, Wang Z, Liu G, Lawlor MW, Moorefield EC, Jones TN, Fugate JA, Furth ME, Murry CE, Ruohola-Baker H, Zhang Y, Santana LF, Childers MK (2014). Dystrophin-deficient cardiomyocytes derived from human urine: new biologic reagents for drug discovery. Stem Cell Res.

[100] Wei R, Han H, Ye M, He L, Lei Q, Zhou T, Cai X, Li Z (2019). Generation of an integration-free iPSC line(SYSUi001-A) from a sporadic Alzheimer's disease patient. Stem Cell Res..

[101] MacDonald WK, Hamilton D, Kuhle S (2014). SMA carrier testing: a meta-analysis of differences in test performance by ethnic group. Prenat Diagn.

[102] Hahnen E, Forkert R, Marke C, Rudnik-Schoneborn S, Schonling J, Zerres K, Wirth B (1995). Molecular analysis of candidate genes on chromosome 5q13 in autosomal recessive spinal muscular atrophy: evidence of homozygous deletions of the SMN gene in unaffected individuals. Hum Mol Genet.

[103] Lefebvre S, Burglen L, Reboullet S, Clermont O, Burlet P, Viollet L, Benichou B, Cruaud C, Millasseau P, Zeviani M (1995). Identification and characterization of a spinal muscular atrophy-determining gene. Cell.

[104] Nash LA, Burns JK, Chardon JW, Kothary R, Parks RJ (2016). Spinal muscular atrophy: more than a disease of motor neurons?. Curr Mol Med.

[105] Monani UR, Lorson CL, Parsons DW, Prior TW, Androphy EJ, Burghes AH, McPherson JD (1999). A single nucleotide difference that alters splicing patterns distinguishes the SMA gene SMN1 from the copy gene SMN2. Hum Mol Genet.

[106] Wirth B, Brichta L, Schrank B, Lochmuller H, Blick S, Baasner A, Heller R (2006). Mildly affected patients with spinal muscular atrophy are partially protected by an increased SMN2 copy number. Hum Genet.

[107] Lotti F, Imlach WL, Saieva L, Beck ES, le Hao T, Li DK, Jiao W, Mentis GZ, Beattie CE, McCabe BD, Pellizzoni L (2012). An SMN-dependent U12 splicing event essential for motor circuit function. Cell.

[108] Ahmad S, Bhatia K, Kannan A, Gangwani L (2016). Molecular mechanisms of neurodegeneration in spinal muscular atrophy. J Exp Neurosci.

[109] Liu H, Lu J, Chen H, Du Z, Li XJ, Zhang SC (2015). Spinal muscular atrophy patient-derived motor neurons exhibit hyperexcitability. Sci Rep.

[110] Simon CM, Janas AM, Lotti F, Tapia JC, Pellizzoni L, Mentis GZ (2016). A stem cell model of the motor circuit uncouples motor neuron death from hyperexcitability induced by SMN deficiency. Cell Rep.

[111] Hor JH, Soh ES, Tan LY, Lim VJW, Winanto SMM, Ho BX, Fan Y, Soh BS, Ng SY (2018). Cell cycle inhibitors protect motor neurons in an organoid model of spinal muscular atrophy. Cell Death Dis..

[112] Cummings BJ, Uchida N, Tamaki SJ, Salazar DL, Hooshmand M, Summers R, Gage FH, Anderson AJ (2005). Human neural stem cells differentiate and promote locomotor recovery in spinal cord-injured mice. Proc Natl Acad Sci U S A.

[113] Salazar DL, Uchida N, Hamers FP, Cummings BJ, Anderson AJ (2010). Human neural stem cells differentiate and promote locomotor recovery in an early chronic spinal cord injury NOD-scid mouse model. PLoS ONE.

[114] Lu P, Wang Y, Graham L, McHale K, Gao M, Wu D, Brock J, Blesch A, Rosenzweig ES, Havton LA, Zheng B, Conner JM, Marsala M, Tuszynski MH (2012). Long-distance growth and connectivity of neural stem cells after severe spinal cord injury. Cell.

[115] Sato M, Takizawa H, Nakamura A, Turner BJ, Shabanpoor F, Aoki Y (2019). Application of urine-derived stem cells to cellular modeling in neuromuscular and neurodegenerative diseases. Front Mol Neurosci.

[116] Kim JY, Chun SY, Park JS, Chung JW, Ha YS, Lee JN, Kwon TG (2018). Laminin and platelet-derived growth factor-bb promote neuronal differentiation of human urine-derived stem cells. Tissue Eng Regen Med.

[117] Geuder J, Wange LE, Janjic A, Radmer J, Janssen P, Bagnoli JW, Muller S, Kaul A, Ohnuki M, Enard W (2021). A non-invasive method to generate induced pluripotent stem cells from primate urine. Sci Rep.

[118] Cong L, Ran FA, Cox D, Lin S, Barretto R, Habib N, Hsu PD, Wu X, Jiang W, Marraffini LA, Zhang F (2013). Multiplex genome engineering using CRISPR/Cas systems. Science.

[119] Pickar-Oliver A, Gersbach CA (2019). The next generation of CRISPR–Cas technologies and applications. Nat Rev Mol Cell Biol.

[120] Wilkinson AC, Dever DP, Baik R, Camarena J, Hsu I, Charlesworth CT, Morita C, Nakauchi H, Porteus MH (2021). Cas9-AAV6 gene correction of beta-globin in autologous HSCs improves sickle cell disease erythropoiesis in mice. Nat Commun.

[121] Kim K, Zhao R, Doi A, Ng K, Unternaehrer J, Cahan P, Huo H, Loh YH, Aryee MJ, Lensch MW, Li H, Collins JJ, Feinberg AP, Daley GQ (2011). Donor cell type can influence the epigenome and differentiation potential of human induced pluripotent stem cells. Nat Biotechnol..

[122] Lee Chong T, Ahearn EL, Cimmino L (2019). Reprogramming the epigenome with vitamin C. Front Cell Dev Biol.

[123] Duan Q, Li S, Wen X, Sunnassee G, Chen J, Tan S, Guo Y (2019). Valproic acid enhances reprogramming efficiency and neuronal differentiation on small molecules staged-induction neural stem cells: suggested role of mTOR signaling. Front Neurosci.

[124] Bershteyn M, Nowakowski TJ, Pollen AA, Di Lullo E, Nene A, Wynshaw-Boris A, Kriegstein AR (2017). Human iPSC-derived cerebral organoids model cellular features of lissencephaly and reveal prolonged mitosis of outer radial glia. Cell Stem Cell.

[125] Faustino Martins JM, Fischer C, Urzi A, Vidal R, Kunz S, Ruffault PL, Kabuss L, Hube I, Gazzerro E, Birchmeier C, Spuler S, Sauer S, Gouti M (2020). Self-organizing 3D human trunk neuromuscular organoids. Cell Stem Cell.

